# Human Cytomegalovirus Encoded Homologs of Cytokines, Chemokines and their Receptors: Roles in Immunomodulation

**DOI:** 10.3390/v4112448

**Published:** 2012-10-25

**Authors:** Brian P. McSharry, Selmir Avdic, Barry Slobedman

**Affiliations:** 1 Discipline of Infectious Diseases and Immunology, University of Sydney, Australia; Email: brian.mcsharry@sydney.edu.au (B.P.McS); selmir.avdic@sydney.edu.au (S.A.); barry.slobedman@sydney.edu.au (B.S.); 2 Centre for Virus Research, Westmead Millennium Institute, Sydney, Australia

**Keywords:** human cytomegalovirus, virus encoded chemokines and cytokines, virus encoded chemokine and cytokine receptors

## Abstract

Human cytomegalovirus (HCMV), the largest human herpesvirus, infects a majority of the world’s population. Like all herpesviruses, following primary productive infection, HCMV establishes a life-long latent infection, from which it can reactivate years later to produce new, infectious virus. Despite the presence of a massive and sustained anti-HCMV immune response, productively infected individuals can shed virus for extended periods of time, and once latent infection is established, it is never cleared from the host. It has been proposed that HCMV must therefore encode functions which help to evade immune mediated clearance during productive virus replication and latency. Molecular mimicry is a strategy used by many viruses to subvert and regulate anti-viral immunity and HCMV has hijacked/developed a range of functions that imitate host encoded immunomodulatory proteins. This review will focus on the HCMV encoded homologs of cellular cytokines/chemokines and their receptors, with an emphasis on how these virus encoded homologs may facilitate viral evasion of immune clearance.

## 1. Introduction

Human cytomegalovirus (HCMV) is one of three β-herpesviruses that infect humans. Following primary (productive) infection, HCMV establishes a life-long latent infection in cells of the myeloid lineage, during which time infectious virus production is undetectable and viral gene expression is highly restricted [1]. Periodically, HCMV can re-activate from latency, resulting in re-initiation of productive infection leading to new, infectious virus. These processes ensure that HCMV infection is widely distributed in the community, with a majority of the world’s population infected by middle age. Whilst primary and reactivated infections are largely asymptomatic or cause only mild disease in immunocompetent individuals, HCMV infection in those who are immunosuppressed or immuno-naïve often leads to life-threatening disease which is difficult to treat or prevent, with complications arising from either primary or reactivated HCMV remaining common in the congenital setting as well as in solid organ and allogeneic stem cell transplant recipients [2]. 

**Figure 1:**
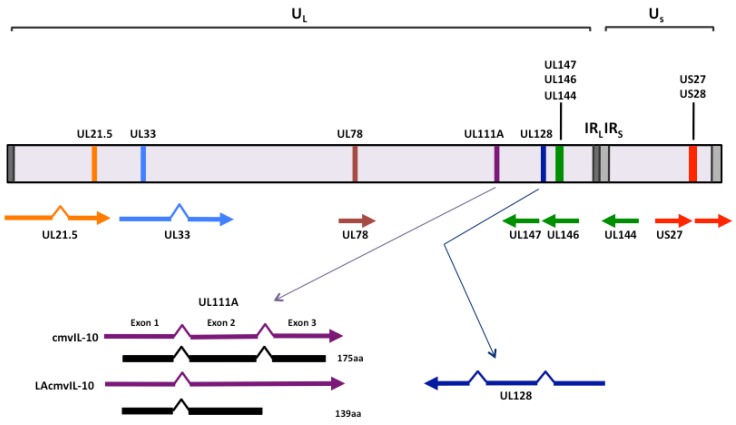
Homologs of cytokines, chemokines and their receptors in the HCMV genome. Schematic representation of the HCMV genome with the positions of the virus encoded homologs of cytokines, chemokines and their receptors indicated. Spliced genes are indicated (^) and the two proteins produced from the UL111A ORF are depicted in black. The terminal and internal repeats flanking the unique long (U_L_) region of the genome (TR_L _and IR_L_, respectively) and the terminal and internal repeats flanking the unique short (U_S_) genome region (TR_S _ and IR_S _, respectively) are also depicted.

**Table 1:** Human cytomegalovirus encoded cytokines, chemokines and their receptors.

Gene name	Homology	Function(s)	Reference(s)
**UL21.5**	Soluble chemokine receptor	Binds CCL5 preventing host cell signaling	*109*
**US27**	Chemokine receptor	Role in extracellular spread of virus	*102*
**US28**	Chemokine receptor	Potential oncogene	*96-99*
		Promotes chemotaxis	*77*
		Potential chemokine sink	*91-93*
**UL33**	Chemokine receptor	Modulates CXCR4 and CCL5 activity	*108*
		Modulates pUS28 activity	*104*
**UL78**	Chemokine receptor	Modulates CXCR4 and CCL5 activity	*108*
		Modulates pUS28 activity	*104*
**UL111A** **(cmvIL-10)**	Cytokine	Inhibits myeloid cell functions	*10-17*
		Stimulates B cell proliferation	*18*
**UL111A** **(LAcmvIL-10)**	Cytokine	Inhibits MHC class II expression	*22*
**UL128**	Chemokine	Promotes PBMC migration	*50*
**UL144**	Cytokine receptor	Inhibits T-cell proliferation via BTLA-4	*38*
		Induces CCL22 via NF-KB	*37, 41*
**UL146**	Chemokine	Promotes neutrophil chemotaxis	*44*
**UL147**	Chemokine	Unknown function	

Despite immunocompetent individuals mounting an enormous, life-long anti-HCMV CD4^+^ and CD8^+^ T cell response [3,4], primary productive infection can persist for an extended period of time, with prolonged shedding of infectious virus in urine and saliva that can last for months in adults and years in children [2]. In addition, this T cell response is ineffective at preventing the establishment of latency or eliminating the latent pool. It has therefore been proposed that HCMV must encode functions which limit host defenses during productive and latent phases of infection. In this respect, studies of viral gene functions have identified a wide range of HCMV gene products with immunomodulatory roles that may enhance the capacity of this virus to productively and/or latently infect the healthy human host. One group of these viral genes shares the common property of having acquired the capacity to mimic cellular cytokines or their receptors. These include chemokines and their receptors, which represent a cytokine subset that mediates chemoattraction. In this review, we summarize current knowledge of HCMV genes that encode homologs of cytokines/chemokines and their receptors (Figure 1 and Table 1), with a focus on how these viral gene products may impair immune clearance of virus during productive or latent phases of infection.

## 2. Cytokine homologs encoded by human cytomegalovirus UL111A

### 2.1 cmvIL-10 and LAcmvIL-10

At present, only one region of the HCMV genome has been shown to code for homologs of cellular cytokines. Initial work by two groups identified a transcript expressed from HCMV UL111A during productive infection as coding for a homolog of human IL-10 (hIL-10), which was termed cmvIL-10 [5,6]. Analysis of HCMV latent infection of myeloid progenitor cells subsequently identified a second hIL-10-like transcript expressed from UL111A, termed latency associated cmvIL-10 (LAcmvIL-10) [7]. In addition to cmvIL-10 and LAcmvIL-10 transcripts, five other transcripts from the UL111A region have been reported to be expressed during the productive phase of infection [8], but as these transcripts have not yet been fully characterized and their biological function not reported, this section on cytokine homologs encoded by HCMV will focus on cmvIL-10 and LAcmvIL-10. 

**Figure 2:**
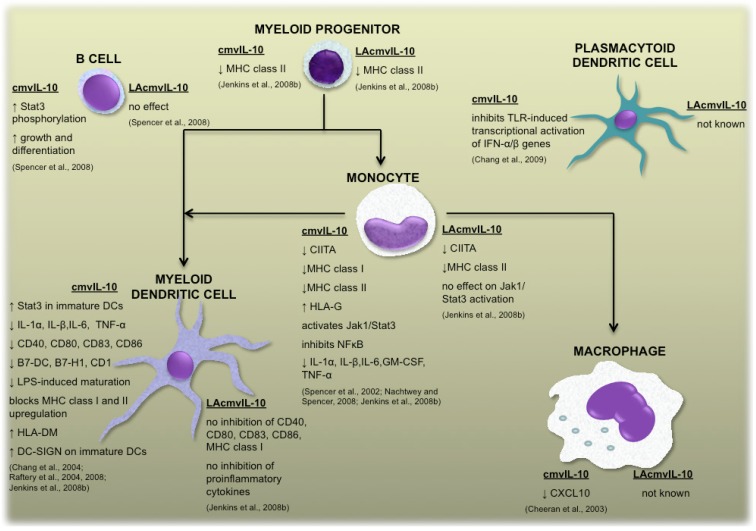
Overview of the functions of cmvIL-10 and LAcmvIL-10. Biological functions of cmvIL-10 and LAcmvIL-10 on immune cells include a range of effects on cellular proteins expressed by myeloid lineage cells during various stages of differentiation/maturation, as well as effects on plasmacytoid dendritic cells and B cells. ↑ indicates upregulation and ↓ indicates suppression.

The cmvIL-10 transcript is comprised of three exons and two introns that encodes a protein of 175 amino acids which shares 27% amino acid identity with hIL-10, but retains the capacity to bind and signal through the hIL-10 receptor [5,6,9]. As a result, the repertoire of cmvIL-10 mediated immunomodulatory functions appears to mimic those of hIL-10 (Figure 2). For example, cmvIL-10 inhibits production of pro-inflammatory cytokines TNF-α, IL-1β and IL-6 in lipopolysaccharide (LPS) stimulated peripheral blood mononuclear cells (PBMCs) and monocytes, and decreases major histocompatibility complex (MHC) class I and MHC class II expression by monocytes [10,11,12]. Aside from inhibition of MHC molecules, cmvIL-10 also inhibits monocyte derived dendritic cell (DC) maturation and inhibits the upregulation of a number of DC costimulatory molecules thus disrupting DC function [13,14,15]. Suppression of pro-inflammatory mediators by cmvIL-10 was also demonstrated in other immune cells, including repressed synthesis of type I interferons by plasmacytoid DCs [16] and suppression of pro-inflammatory CXC chemokine ligand 10 (CXCL10) by microglial macrophages, resulting in limited chemotaxis of activated T lymphocytes towards these cells [17]. 

Apart from these immunosuppressive properties manifested on cells of myeloid origin, cmvIL-10 has been shown to stimulate proliferation and differentiation of B lymphocytes [18] and monocyte differentiation towards a phagocytic phenotype [19]. In addition, a role for cmvIL-10 has also been suggested in enhancing congenital HCMV disease by impairing cytotrophoblast remodeling of the uterine vasculature to limit fetal growth, as it has been demonstrated that cmvIL-10 decreased matrix metalloproteinase activity in endothelial cells and cytotrophoblasts and could impair endothelial cell migration and cytotrophoblast invasiveness *in vitro* [20]. This combination of cmvIL-10 controlled suppression of pro-inflammatory mediators and other important cellular functions, accompanied by stimulation of B cells may work together to tilt the host immune response towards a phenotype that is less effective at controlling virus replication. This may enhance the capacity of HCMV to not only more effectively establish primary productive infection, but may also contribute to chronic productive infection at sites of persistent viral shedding.

The LAcmvIL-10 transcript was initially identified during HCMV latency, and is a variant of the cmvIL-10 transcript [7]. Subsequent analysis has revealed that the LAcmvIL-10 transcript is also expressed during productive infection of human fibroblasts [21]. Both the site of initiation of translation and the transcriptional termination site are predicted to be the same as that of cmvIL-10, however differential splicing of the LAcmvIL-10 transcript results in the second intron of the cmvIL-10 mRNA being incorporated into the LAcmvIL-10 mRNA, resulting in an in-frame stop codon. As a consequence, the LAcmvIL-10 protein is predicted to be identical with the cmvIL-10 protein for the first 127 amino acids but then diverges for its final 12 amino acids at the C terminus, resulting in a truncated protein of 139 amino acids [7]. Nonetheless, like cmvIL-10, LAcmvIL-10 shares 27% identity with hIL-10.

Whilst cmvIL-10 exerts a broad range of immunomodulatory properties, the function of LAcmvIL-10 appears to be much more limited (Figure 2). Both cmvIL-10 and LAcmvIL-10 suppress MHC class II by primary human myeloid progenitor cells and monocytes [12,22]. However, unlike cmvIL-10 which impairs DC maturation [13,14,22], LAcmvIL-10 does not possess this function, as indicted by a lack of capacity to inhibit expression of proinflammatory cytokines TNF-α, IL-1α, IL-1β and IL-6 and costimulatory molecules CD40, CD80, and CD86 by DC stimulated to mature with LPS [22]. Furthermore, whilst cmvIL-10 stimulates B cells and phagocytic macrophages, LAcmvIL-10 does not [18,19]. The basis of the differential functional effects of cmvIL-10 and LAcmvIL-10 has not been fully elucidated, but the structural differences between these two viral cytokines provide some insights. In particular, it has been shown that whilst cmvIL-10 binds and signals through the hIL-10 receptor in a manner comparable to that of hIL-10, LAcmvIL-10 appears to exert its function differently. Studies using antibody to neutralize the hIL-10 receptor and analysis of cellular signaling events triggered by engagement of the hIL-10 receptor have demonstrated that LAcmvIL-10 either does not bind and signal through the hIL-10 receptor, or it does so in a manner different to that of cmvIL-10 and hIL-10 [22]. It therefore remains to be determined whether LAcmvIL-10 modulates MHC class II via engagement of a different cellular receptor, or whether it binds to the hIL-10 receptor in a unique manner which results in a restricted repertoire of functions.

Studies of the functions of cmvIL-10 and LAcmvIL-10 have mostly relied upon the use of recombinant proteins synthesized either from bacterial or mammalian expression systems. However, several studies have examined biological function in the context of productive or latent infections utilizing UL111A deletion viruses unable to express both cmvIL-10 and LAcmvIL-10, and collectively referred to as viral IL-10 (vIL-10) deletion viruses. Deletion of the vIL-10 gene from HCMV does not affect virus replication during *in vitro* HCMV productive infection of human fibroblasts [23], nor does it impinge upon the establishment and maintenance of HCMV latency *in vitro* [24]. However, vIL-10 acts to actively restrain the host’s immune response against HCMV during experimental latent infection, as shown by suppression of surface MHC class II on latently infected cells with accompanied suppression of CD4^+^ T cell recognition of latently infected cells [24]. Furthermore, in comparison to parental virus (with intact UL111A), vIL-10 deletion virus upregulates cytokines associated with DC formation during experimental latency and increases the proportion of myeloid DCs, some of which are Langerhans cells [25]. Thus, expression of vIL-10 during latency modifies differentiation of latently infected myeloid progenitors to inhibit formation of DCs, and this has been suggested to represent an additional means by which HCMV may restrict immune clearance of latently infected cells. It is not yet clear whether the inhibition of differentiation to a DC is due to vIL-10 mediated blockade of myeloid cell differentiation, as opposed to a skewing of differentiation to a different, less immunogenic myeloid cell type. Our hypothesis is that vIL-10 expressed during HCMV infection promotes an anti-inflammatory macrophage phenotype to aid in virus pathogenesis and this is a current focus of broader analyses of vIL-10 function in the context of HCMV infection. 

### 2.2 Vaccine design based upon the functions of human cytomegalovirus-encoded IL-10

Due to the ability of HCMV to cause severe or fatal disease in high risk groups such as congenitally infected fetuses and immunocompromised individuals, and the corresponding high health care costs associated with their treatment and care, development of an HCMV vaccine has been made a top priority by the Institute of Medicine in the US [26]. However, the quest for a licensed HCMV vaccine remains unfulfilled, despite a number of attempts over the last half a century (for comprehensive reviews of the HCMV vaccine field, see [27,28]).

It could be argued that the immunomodulatory properties of HCMV encoded vIL-10 proteins may represent a potential problem for the development of any live attenuated HCMV vaccine candidate as the host’s immune response towards such a vaccine may be suboptimal due to the expression of vIL-10 (present in all laboratory and wild type strains sequenced so far), resulting in decreased production of host’s pro-inflammatory cytokines and chemokines that drive the recruitment of immune cells towards the site of vaccination and/or suppression of DC maturation and limited antigen presentation due to decreased levels of MHC. Thus, the functions of vIL-10 should be considered in the development of such a vaccine to prevent HCMV disease. 

In this respect, recent attempts have been made to engineer a live vaccine virus based upon deletion of a CMV encoded IL-10 homolog. Due to its strict species specificity, these investigations have, to date, relied upon the use of an animal model of HCMV infection, using rhesus CMV (RhCMV) [29,30]. Unlike murine CMV (MCMV), RhCMV encodes its own functional IL-10 homolog (RhcmvIL-10) [6]. RhCMV infection of rhesus macaques in many aspects mirrors natural HCMV infection [31] and this model enables vaccination-challenge experiments to be undertaken. The significance of CMV encoded IL-10 function *in vivo* is emphasized by a recent study that compared the effects of wild type RhCMV to the RhcmvIL-10 deletion mutant. In comparison to infection with wild-type RhCMV, rhesus macaques infected with a RhcmvIL-10 deletion virus displayed increased innate and adaptive immune responses, characterized by a higher inflammatory response, decreased macrophage infiltration but a significant increase in DC numbers and stronger CD4^+^ T cell proliferation, as well as higher IgG titers [30]. 

In addition to live vaccine design based upon deletion of RhcmvIL-10, a DNA vaccine encoding for RhcmvIL-10 protein has also been explored. Whilst this initial approach resulted in relatively weak immunogenicity [32], during natural RhCMV infection, RhcmvIL-10 was found to be highly immunogenic, resulting in strong induction of anti-RhcmvIL-10 antibodies in plasma that were capable of neutralizing RhcmvIL-10 function without cross reactivity with rhesus cellular IL-10, with anti-RhcmvIL-10 specific T cells identified in 22% of infected animals [33]. In the context of HCMV infection, antibody responses have also been detected against cmvIL-10 in 28% of infected individuals [34]. Anti-cmvIL-10 antibodies also demonstrated potent neutralizing function exclusively against cmvIL-10, with no cross reactivity with either hIL-10 or Epstein-Barr virus encoded IL-10 (ebvIL-10) [34].

To further investigate RhcmvIL-10 as a surrogate vaccine candidate for vIL-10 encoded by HCMV, mutated versions of RhcmvIL-10 unable to bind to the IL-10R but still preserving tridimensional structure of wild type RhcmvIL-10 were generated [29]. Although these mutated RhcmvIL-10 proteins lacked immunomodulatory functions of wild type RhcmvIL-10, they generated a strong anti-RhcmvIL-10 antibody response in infected animals that received DNA vaccines encoding these mutated forms of RhcmvIL-10. Importantly, these antibodies were capable of blocking the biological activity of wild type RhcmvIL-10 and did not cross react with cellular rhesus IL-10 [29]. In addition, immunization of seronegative animals with mutated forms of RhcmvIL-10 prior to infection with RhCMV resulted in prominent decrease in the number of cells infected with RhCMV as well as the frequency and amount of detectable RhCMV in these animals compared to mock immunized animals [35]. 

Whilst this review focuses on HCMV encoded cytokines/chemokines and their receptors, these studies of RhcmvIL-10 highlight the biological relevance of CMV encoded IL-10 *in vivo* and also provide an excellent example of how functional analyses of HCMV encoded IL-10 *in vitro* have helped to inform the development of novel potential vaccine strategies. 

## 3. Cytokine receptor homolog encoded by the human cytomegalovirus UL144 gene

The protein product of the UL144 gene was originally identified as having homology with tumor necrosis factor receptor (TNFR) superfamily members based on its similarity with the herpesvirus entry mediator, HVEM, encoded by herpes simplex virus type 1 (HSV-1) [36]. UL144 is an early gene that encodes a type I transmembrane glycoprotein that remains predominantly intracellular [36]. It does not appear that the UL144 protein (pUL144) functions on infected cells through binding of TNF ligand family members as screening by both ELISA [36] and surface plasmon resonance [37] with panels of such molecules was not able to detect direct binding. However, a potential function for pUL144 in modulating the host immune response was demonstrated using an Fc fusion protein of the extracellular domain of UL144 [38]. This truncated fusion protein was capable of binding to the immunoglobulin family member BTLA (B- and T-lymphocyte attenuator), an inhibitory receptor found on T-cells [39]. This interaction blocked CD4^+^ T-cell proliferation induced by anti-CD3 and anti-CD28 stimulation in a manner analogous to HVEM, however unlike HVEM the UL144 fusion protein was not capable of binding to the T-cell activating receptor, lymphotoxin-like inducible protein that competes with glycoprotein D for herpesvirus entry on T cells (LIGHT) [38]. Such a function could have obvious beneficial effects for the virus in modulating the potent anti-viral T-cell response detected *in vivo*. However, these effects were only demonstrated using a recombinant fusion protein and it would be important to determine if virally expressed pUL44 can elicit similar effects on T-cell activation following HCMV infection.

pUL144 can also act within the infected cell to induce NF-κB activation and modulate host gene expression without the requirement for ligand binding [37]. pUL144 is capable of activating NF-κB expression when expressed in isolation or in the context of HCMV infection. This effect was TNFR associated factor 6 (TRAF6) dependent [37] and also requires the cellular tripartite motif 23 protein (TRIM23) as a co-factor to potentiate signaling [40]. Both TRIM23 and TRAF6 can directly interact with pUL144 [37,40] and it has been postulated that formation of a complex of TRAF6, TRIM23 and pUL144 is necessary to induce NF-κB signaling [40]. Screening of infected cells for cytokines and chemokines modulated by pUL144 expression identified the chemokine CCL22 as induced by viruses containing the UL144 ORF and was specifically inhibited using siRNA targeting the UL144 transcript [37,41]. The CCL22 chemokine is a known chemoattractant of regulatory T-cells [42] and it has been suggested that this is how UL144 may act as an immunoevasin [37]. However there has been no direct evidence to date that UL144 expression by HCMV can attract regulatory T-cells and so further study will be required to demonstrate such a function.

## 4. Chemokine homologs encoded by human cytomegalovirus *UL146*, *UL147* and *UL128*

Chemokines are a subset of cytokines that are able to induce chemotaxis of target cells that express a cognate seven-transmembrane G protein-coupled chemokine receptor. There are four groups of chemokines, based upon the arrangement of the first two N-terminal cysteine residues; CC, CXC, C and CX3C chemokines [43]. CXC and CC chemokines are also known as α and β chemokines, respectively, and are involved in a diverse range of functions to control immune and inflammatory responses. To date, three HCMV encoded homologs of CXC or CC chemokines have been reported; UL146, UL147 and UL128. 

### 4.1 UL146 and UL147

HCMV encodes two genes, UL146 and UL147, whose protein products exhibit limited identity with CXC-chemokines [44]. This homology includes putative signal sequences, cysteine spacing and for pUL146 an ELR-CXC sequence (glutamic acid, leucine, arginine [ELR] upstream of the CXC motif) that is common in a number of CXC-chemokines, including IL-8 and Gro-α. HCMV strain Toledo UL146 and UL147 exhibit 24% and 16% amino acid identity, respectively, to IL-8. ELR-CXC chemokine family members are known to play important roles in neutrophil metabolism [45]. Although the virus encodes two potential homologs of CXC chemokines, to date only the functional effects of pUL146 (vCXCL-1) have been reported in any detail. 

Disruption/deletion of the UL146 gene from the HCMV strain Toledo genome limited the ability of HCMV infected fibroblasts to promote neutrophil chemotaxis. In addition, bacterially expressed pUL146 could also induce chemotaxis of human neutrophils to similar levels as IL-8 and Gro-α [44]. It was suggested that pUL146 mediated its effects through binding of CXCR2, rather than CXCR1, based on stable cell lines expressing each of the chemokine receptors, and could compete with IL-8 for binding to CXCR2 [44]. A more recent study examined the ability of pUL146 to activate signaling from a panel of 18 defined human chemokine receptors and demonstrated via both chemotaxis and calcium mobilization that pUL146 could induce signaling through both CXCR1 and CXCR2 [46], although the binding of pUL146 to CXCR1 was of lower affinity and potency. It would seem counterintuitive that HCMV should encode a function to recruit immune effectors such as neutrophils, however it has been postulated that neutrophils could act as potential passive carriers of HCMV to facilitate virus dissemination [44,46], providing at least a hypothetical role for pUL146 in enhancing virus spread. 

### 4.2. UL128

The UL128 ORF encodes a spliced transcript whose protein product forms part of a pentameric complex with pUL130, pUL131A, gH (pUL115) and gL (pUL75) that plays a vital role in mediating entry of HCMV into both epithelial and endothelial cells [47]. Genes in the UL128 locus (UL128, UL130 and UL131A) are rapidly mutated following viral propagation in human fibroblasts but are maintained following passage in endothelial/epithelial cells [48]. In addition to its characterized role in HCMV entry, the UL128 protein (pUL128) contains four conserved cysteine amino acids at its N-terminus that are characteristic of the motif found in CC-chemokines [49]. On the basis of this motif it has been proposed that pUL128 may act as a chemokine [49]. Most recently, Zheng *et al*. expressed a soluble version of pUL128 and showed that it was capable of promoting migration of human PBMC to a similar level as another CC-chemokine MIP-1α/CCL3. Treatment of human PBMC with the soluble UL128 protein (pUL128) also induced increased expression of the pro-inflammatory cytokines TNF-α and IL-6 [50] and it is known that CC-chemokines can induce such cytokines [51]. How pUL128 promotes such activity, if it is related to its CC chemokine-like structure and if it is capable of signaling via CC-chemokine receptors has not yet been reported. 

## 5. The chemokine homolog UL146 and the cytokine receptor homolog UL144 are hypervariable genes

Both UL144 and the UL146 are located in the UL/b’ region of the HCMV genome; a region that has been lost in highly passaged laboratory strains [52]. In addition, both UL144 and UL146 are among a limited number of hypervariable genes encoded by HCMV [53,54]. UL146 exhibits less than 40% amino acid identity across genotypes and UL144 exhibits variation in more than 30% of amino acid positions [53,54]. A number of attempts have been made to link UL144 and UL146 polymorphisms with clinical disease, primarily in the congenital disease setting. Most reports have not identified an association between UL144 genotype and HCMV congenital disease/sequelae [55,56,57,58,59], however some studies have provided evidence of particular UL144 genotypes being associated with clinical outcome [60,61]. Similar studies have also looked at UL146 sequence variability and clinical disease without significant association [55,62,63]. However, the use of artificial neural networks (statistical modeling programs used to find patterns between data) to associate UL144 and UL146 sequence variability with clinical outcome was successful for both UL144 (80% of cases) and UL146 (85% of cases) [64]. Additional studies examining larger cohorts may help resolve the differences reported to date. Interestingly, no significant functional differences between UL144 and UL146 variants have been identified in *in vitro* studies to date [37,38,63] despite the high levels of variation between genotypes and the physiological relevance (if any) of such differences remains to be elucidated. 

## 6. Chemokine receptor family members encoded by human cytomegalovirus

G-protein coupled receptors (GPCRs) play a crucial role in the cellular response to a wide variety of external stimuli including hormones, neurotransmitters and chemokines [65]. Chemokine receptors share a common structure consisting of a seven transmembrane motif coupled to heterotrimeric G-proteins to mediate intracellular signaling. Chemokine binding to its cognate receptor can activate a wide range of downstream signaling pathways in host cells including the MAP (mitogen-activated protein) kinase, phospholipase C, Rho GTPases and phosphoinositide 3-kinase (PI 3-kinase) cascades leading to diverse effects on host cell metabolism and trafficking [66].

Following complete sequencing of the HCMV strain AD169 genome it was recognized that HCMV encodes a family of seven transmembrane proteins that have homology to chemokine receptors [67]. Three HCMV encoded genes: US27, US28 and UL33 were recognized to encode such motifs (Chee 1990) before a fourth member of this family, UL78 was identified subsequently based on homology with a human herpes virus-6 (HHV-6) protein, U51 [68]. By far the best studied member of this family of viral proteins is pUS28 which can function as a true chemokine receptor and has been implicated in a variety of important roles in the viral life cycle.

### 6.1. US28

A number of publications have reviewed US28 functions [69,70,71] and a complete discussion of the pleiotropic roles of US28 is beyond the scope of this review. Here, we will focus on more recent reports detailing US28 function as a potential oncogene as well its potential immunomodulatory role during viral infection. 

US28 is an early (E) gene with mRNA detected as early as 2 hours post infection in human fibroblasts [72]. US28 exhibits 30% homology with the β-chemokine receptor CCR1 and also exhibits homology in its ligand binding domain for other chemokine receptors including CCR2 and CCR5 [73]. pUS28 is the only viral family member that has been shown to bind chemokines, and can act as a receptor for a wide range of chemokines including the CC chemokines RANTES/CCL5, MCP-1/CCL2, MCP-3/CCL7, MIP-1α/CCL3 and MIP-1β/CCL4 as well as the CX3C chemokine, fractalkine/CX3CL1 [73,74,75]. Chemokine binding to pUS28 can induce an array of cellular responses including MAP kinase activation, calcium flux, cell migration and activation of transcription factors such as cyclic AMP responsive element binding protein (CREB) and NF-κB [73,76,77,78,79]. pUS28 also displays agonist independent, constitutive signaling activity inducing phospholipase C activation leading to production of the second messengers, inositol triphosphate and diacyl glycerol, and as well as promoting NF-κB activation [80,81,82,83,84]. Through such signaling pathways pUS28 can induce a wide variety of effects on host cell metabolism including promoting smooth muscle cell migration [76], which has been implicated in the association between HCMV infection and atherosclerosis [85]. A recent study also demonstrated that US28 from HCMV can be functional following viral expression in an *in vivo* setting [86]. Deletion of the UL33 homolog (M33), encoded by MCMV, from the viral genome limits salivary gland replication and is associated with reduced reactivation from spleen and lung explants [86,87]. Replacing M33 with US28 from HCMV at least partially complements the activity of M33 [86] and provides a system to study US28 activity in an animal model.

Essentially all known signaling functions of the protein have been first demonstrated using pUS28 expressed in isolation with much more limited numbers of reports demonstrating/confirming such activities in the context of a productive HCMV infection. It is also possible to detect US28 transcription following HCMV infection in a number of different models of latency [88,89,90] although the potential role of US28 expression in this setting has not yet been elucidated.

#### 6.1.1. US28 immunomodulation

It has been postulated that pUS28 can act as an immunoevasin, primarily based on the ability of the protein to bind to and internalize a range of chemokines, thus limiting the ability of such chemotactic molecules to elicit their effects on surrounding immune cells. Bodaghi *et al*. first demonstrated that HCMV infected fibroblasts could bind the chemokines CCL2, CCL7, CCL4 and CCL5 (including CCL5 and CCL2 produced from infected cells) leading to a reduction in extracellular accumulation of these molecules [91]. This chemokine binding activity of infected cells was dependent on viral expression of pUS28. In addition to infected cells binding extracellular chemokines, a number of studies have reported that CCL5 can be internalized into both infected fibroblasts and endothelial cells by pUS28 [91,92]. It has been shown that CCL5 and CCL2 expression, detectable following infection with a HCMV strain Towne US28 deletion mutant, could induce monocyte chemotaxis that was inhibited by infection with the parental strain expressing US28 [93]. However, the chemokine sequestering activity of retrovirally expressed pUS28 was not sufficient to block adhesion of leucocytes to TNFα activated endothelial cells [94]. 

#### 6.1.2. US28 in tumorigenesis

Over the last ten years there have been an increasing number of reports linking HCMV infection with human malignancies, in particular, glioblastoma [95]. There have also been publications providing evidence of a potential role for US28 in promoting tumorigenesis [96,97,98]. NIH-3T3 murine fibroblasts engineered to express pUS28 exhibited a number of properties consistent with a transformed phenotype including increased growth, enhanced cell cycle progression and expression of the pro-angiogenic factor vascular endothelial growth factor (VEGF) [96] as well as cyclooxygenase-2 (COX-2), an enzyme associated with a range of human tumors [97]. The proliferative activity of cells expressing pUS28 was associated with upregulation of STAT3/IL-6 via NF-κB activation induced by US28 [98]. When pUS28 positive NIH-3T3 cells were injected into a nude mice model, tumors formed much more rapidly than following injection of the parental cells or cells expressing a US28 mutant that lacks constitutive signaling activity, implicating this activity in oncogenesis [96,97]. The ability of pUS28 to induce VEGF, COX-2, and phosphorylated STAT3 was also demonstrated in the context of HCMV infection [96,97,98]. A transgenic mouse model expressing pUS28 under the control of the villin promoter which targeted expression to intestinal epithelial cells has also been investigated [99]. Mice expressing the transgene developed intestinal neoplasia that was linked with dysregulation of a number of genes controlling cell proliferation, including survivin and cyclin-D1 and was further enhanced by co-expression of the US28 ligand, CCL2 [99]. US28 transcript and protein can also be detected in cultures of primary glioblastomas and from paraffin embedded glioblastoma tissue samples in approximately 60% of cases analyzed [98,100].

### 6.2. US27, UL33 and UL78

Compared to US28 relatively little is known about the function of the three other virally encoded members of the 7 transmembrane family, and to date these are orphan receptors with no known chemokine ligands. However, recent publications may provide insights into how US27, UL33 and UL78 may function as they appear to heteromerize with both viral and cellular G-protein coupled receptors modulating their function. 

#### 6.2.1. US27

pUS27 is expressed with late gene kinetics, but unlike the other family members, pUS28 and pUL33, does not appear to possess constitutive signaling activity and no activating ligand has been identified to date [101]. pUS27 is predominantly located intracellularly but can be detected at the cell surface and is incorporated into the virion envelope [101,102,103]. The study of mutant viruses that lack US27 expression in both the FIX and TB40E strain backgrounds have indicated that pUS27 appears to play a role in extracellular spread of the virus in both fibroblasts and endothelial cells with US27 mutants producing 10-fold less extracellular virus compared to the parental strains [102]. Direct cell to cell spread of the virus was not affected in these mutants. How pUS27 can promote extracellular accumulation of virus and whether it relates to its GPCR-like structure remains to be elucidated. pUS27 can also colocalize and heteromerize with pUS28 but does not appear to inhibit pUS28 mediated NF-κB activation or inositol phosphate accumulation [104] and the role of this pUS27-pUS28 complex is as yet unknown. 

#### 6.2.2. UL33 and UL78

The UL78 protein is encoded by an early gene that is non-essential for replication *in vitro* [105]. The UL33 gene is also expressed with early gene kinetics, encodes an N-linked glycoprotein and is non-essential for growth in human fibroblasts [106]. Both UL78 and UL33 have homologs in both simian and murine cytomegaloviruses, suggesting an important role for these proteins in the viral life cycle. No ligand for either pUL33 or pUL78 has yet been identified. pUL33 does exhibit constitutive signaling activity, activating the transcription factor, CREB when expressed in isolation or in the context of viral infection and is also capable of inducing inositol triphosphate accumulation through the phospholipase C pathway but is not capable of inducing NF-κB activity [81,82].

A number of recent publications have indicated that pUL78 and pUL33 are capable of heteromerizing with other G-protein coupled receptors. Two recent reports demonstrated that both pUL78 and pUL33 can form a complex with pUS28 [104,107] but this did not affect the ability of US28 to signal through phospholipase C to induce inositol triphosphate. However, it was reported that such complex formation can inhibit the ability of pUS28 to induce NF-κB activation suggesting a potential regulatory role in controlling signaling in infected cells [104]. These publications have only demonstrated such effects in transient transfection systems and it will be important to confirm this activity in the context of viral infection.

In addition to interacting with the virally encoded G-protein coupled receptor, pUS28, it has also been shown that both pUL78 and pUL33 can heteromerize with the chemokine receptors CCR5 and CXCR4 in myeloid lineage THP-1 cells [108]. This heteromerization led to changes in a number of important biological activities of these chemokine receptors including an inhibition of HIV-1 infection of THP-1. Cells expressing either pUL33 or pUL78 became resistant to HIV-1 infection, presumably through modulation of the HIV co-receptor activity of CCR5 and CXCR4 [108]. In addition, expression of pUL33 and pUL78 can modulate the response to chemokines that bind to CCR5 and CXCR4, including CCL5 and CXCL12, thus regulating the chemotactic activity induced by ligand binding [108]. These effects were demonstrated using viral proteins expressed from plasmid vectors and it will important to investigate such responses in the context of viral infection. Nonetheless, these studies represent important insights into how the HCMV-encoded orphan chemokine-like receptors of previously unknown function may modulate the host immune response.

### 6.3. UL21.5

pUL21.5 (also known as pUL22A) is a small secreted glycoprotein encoded by HCMV that is dispensable for growth of both laboratory strains and clinical isolates in human fibroblasts [109]. Screening of a small panel of chemokines identified that pUL21.5 is capable of binding to the CC chemokine CCL5 (RANTES) but was unable to bind two other CC chemokines; CCL3 and CCL2 or to the CXC chemokine IL-8 [109]. Thus pUL21.5 may act as a sink to sequester free RANTES and is capable of competing for CCL5 binding to cell surface receptors on THP-1 cells [109]. Interestingly the mRNA encoding pUL21.5 is one of a limited subset of viral encoded RNAs that are incorporated into the mature virion [110] and thus should be available for translation immediately following infection without the requirement for *de novo* gene transcription. 

CCL5 is a potent chemoattractant of immune effector cells such as monocytes and T-cells that bear the CCR5 receptor and can also signal through both CCR3 and CCR1 to recruit other cells including both macrophages and dendritic cells [111]. HCMV encodes at least five independent functions to modulate CCL5 expression and function, strongly suggesting an important role for this chemokine in the anti-HCMV immune response. In addition to pUL21.5, pUS28 has also been shown bind CCL5 potentially further sequestering this chemokine [73,92]. CCL5 mRNA transcription is induced by infection with UV irradiated HCMV that can be inhibited by expression of the IE86 immediate early protein following productive infection [112]. HCMV also encodes a miRNA, mir-UL148D, not expressed by laboratory strains of the virus, that can target CCL5 mRNA for degradation at late times post infection [113]. In addition, both pUL33 and pUL78 can modulate the response to CCL5 by modulating signaling through CCR5 (see section 6.2.2).

## 7. Conclusions

Pirating of host encoded proteins provides pathogens with a means to exploit existing cellular signaling pathways to facilitate infection, dissemination and maintenance through immune evasion. HCMV encodes multiple proteins that have homology to cytokines, chemokines and receptors providing the virus with an arsenal of functions to counteract the host immune response, given the central role such molecules play in controlling immune activity. The virally encoded homologs described here share some activities with their cellular counterparts but also demonstrate actions that have presumably emerged through viral evolution e.g. constitutive or ligand independent signaling of chemokine receptors allowing further subversion of the anti-viral host response. Whilst studies detailed here have provided many important insights into viral immunomodulatory activity, it is clear that further study is required to fully appreciate the role these HCMV genes can play. In particular, a majority of these reports have used heterologous protein expression systems in cell lines *in vitro* to study gene function, so it will be important to build on these studies to examine the gene’s role(s) in the context of viral infection as well as in more physiological relevant cell types and *in vivo*. In this respect, the much enhanced capacity to rapidly generate viral gene deletion/mutant viruses, together with a greater understanding of (i) models of HCMV infection of different permissive primary human cell types and (ii) animal models of infection with other cytomegaloviruses (e.g. murine CMV and rhesus CMV), are likely to significantly enhance our capacity to more fully define the functional impacts of these virus encoded immune modulators.
